# Development and preliminary evaluation of an oral health training program for diabetes educators: a quasi-experimental study

**DOI:** 10.3389/froh.2026.1819829

**Published:** 2026-05-21

**Authors:** Ajesh George, Ariana Kong, Prakash Poudel, Amit Arora, Phyllis Lau, Rhonda Griffiths, Angela Masoe, Susanne Sofronoff, Rachel E. Martin, Lorena Akerman, Jennifer Wong, Natalia Uthurralt, Andre Priede, Vincent W. Wong, Cathryn Forsyth, Parul Marwaha, Shwetha Kezhekkekara, Hanny Calache

**Affiliations:** 1 Australian Centre for Integration of Oral Health (ACIOH), School of Nursing & Midwifery, Western Sydney University, Liverpool, NSW, Australia; 2 Ingham Institute for Applied Medical Research, Liverpool, NSW, Australia; 3 School of Dentistry, Faculty of Medicine and Health, The University of Sydney, Surry Hills, NSW, Australia; 4 Faculty of Science, Medicine and Health University of Wollongong, Wollongong, NSW, Australia; 5 Manipal College of Dental Science, Manipal Academy of Higher Education, Mangalore, India; 6 Translational Health Research Institute, Western Sydney University, Campbelltown, NSW, Australia; 7 School of Health Sciences, Western Sydney University, Campbelltown, NSW, Australia; 8 Health Equity Across Lifespan (HEAL) Research Laboratory, Campbelltown, NSW, Australia; 9 Discipline of Child and Adolescent Health, Sydney Medical School, The University of Sydney, Campbelltown, NSW, Australia; 10 Sydney Dental Hospital, Sydney Local Health District, Surry Hills, NSW, Australia; 11 Melbourne Dental School, The University of Melbourne, Carlton, VIC, Australia; 12 School of Medicine, Western Sydney University, Campbelltown, NSW, Australia; 13 Department of General Practice, The University of Melbourne, Melbourne, VIC, Australia; 14 NSW Ministry of Health, Centre for Oral Health Strategy, St Leonards, NSW, Australia; 15 Oral Health Victoria, Carlton, VIC, Australia; 16 Oral Health Services, Barwon Health, Newcomb, VIC, Australia; 17 Australian Diabetes Educators Association, Turner, ACT, Australia; 18 School of Clinical Sciences, Monash University, Clayton, VIC, Australia; 19 School of Clinical Sciences, Monash University, Clayton, VIC, Australia; 20 South-Western Sydney Clinical School, University of New South Wales, Liverpool, NSW, Australia; 21 Fairfield & Liverpool Diabetes Services, South Western Sydney Local Health District, Liverpool, NSW, Australia; 22 Dental Services, Monash Health, Springvale, VIC, Australia; 23 School of Health and Social Development, Faculty of Health, Institute for Health Transformation, Deakin University, Burwood, VIC, Australia; 24 La Trobe Rural Health School, La Trobe University, Bendigo, VIC, Australia

**Keywords:** diabetes, diabetes educators, health promotion, oral health, periodontal disease

## Abstract

**Background:**

Although diabetes and oral health share a bidirectional relationship, there is little emphasis on oral health promotion by diabetes care providers despite global guidelines and recommendations. In Australia, diabetes educators are well-placed to promote oral health among their clients; however, comprehensive training programs tailored for diabetes educators are lacking.

**Aims:**

To develop and evaluate a diabetes oral health training program to assess the potential impact in improving oral health knowledge and confidence of diabetes educators, and the acceptability, feasibility, and sustainability of integrating the training into practice.

**Methods:**

Guided by the Medical Research Council framework for complex interventions, a mixed-methods, quasi-experimental one-group pretest-posttest design was used. The development led to a three-module training program, which integrated videos, a screening tool, and resources to support oral health referrals. A pre-post knowledge and confidence questionnaire was analysed using a paired-sample t-test. To assess acceptability, feasibility and sustainability, qualitative semi-structured interviews were conducted and analysed using directed content analysis.

**Results:**

Thirty diabetes educators from the states of New South Wales and Victoria were recruited. There was an overall significant difference in the knowledge scores pre-test (mean = 23.24, SD 4.4) compared to post-test (mean = 29.67, SD 2.01), (t = −6.11), *p* < 0.001) (*n* = 21), and across all confidence variables on discussing, screening and referring clients to oral health services (*p* < 0.001). Most participants agreed with the trainings perceived acceptability, feasibility, and sustainability, although some recommendations were made to improve long-term sustainability.

**Conclusions:**

Using online training modules and tailored resources to support clinical practice has shown preliminary improvements in oral health knowledge and confidence among diabetes educators, and was acceptable, feasible, and sustainable for integration into clinical practice.

## 1 Introduction

More than an estimated 828 million adults worldwide have diabetes (1). While trends indicate that the greatest proportional increases have been in low to middle income countries, this increase represents an overall increase of more than 630 million people since 1990 (1, 2). Diabetes is a factor in the development of many other diseases including chronic kidney disease, cardiovascular disease including stroke and ischaemic heart disease. This results in a huge economic burden to the health system; for example, one study estimated a projected increase in global economic burden of US$2.1 trillion by 2030 (3).

Diabetes can lead to various oral health complications (dry mouth, tooth loss, oral thrush, and taste impairment caries, periodontitis, etc.), significantly affecting the mouth's overall condition. Periodontal (gum) disease is identified as the sixth most common complication of diabetes, following micro- and macrovascular complications (4). Diabetes and periodontal disease have a bidirectional relationship; diabetes increases the risk of periodontal disease, and periodontal disease negatively affects glycaemic management, and in turn increases the risk of diabetes complications (5). Chronic periodontal disease (periodontitis) may have profound negative impacts on various aspects of daily living and quality of life, affecting confidence, social interactions, and food choices.

Periodontal treatment (PT), including deep cleaning, scaling, root planning, has demonstrated significant improvements in glycaemic management, reducing glycated haemoglobin (HbA1c) level by 0.43%, thus managing type-2 diabetes in three to four months (6). The International Diabetes Federation and European Federation of Periodontology published guidelines recognising the importance of oral health care in diabetes management and recommend that diabetes care providers undertake an oral health review of people with diabetes (7). Similar guidelines underscoring the need for general practitioners (GPs) to conduct oral health reviews for people with diabetes have been published in Australia (8). Despite these guideline recommendations, there appears to be limited emphasis on oral health promotion by diabetes care providers, including GPs and diabetes educators (DEs) in Australia, with the main barriers being limited knowledge and training in this area (9, 10). Globally people with diabetes have also reported poor oral health knowledge and have received only limited oral health information from diabetes care providers (11). These findings highlight the need to improve the oral health competency and confidence of diabetes care providers to promote oral health.

DEs are at the forefront of preventing and managing diabetes in public health services (12). Despite clinical practice guidelines recommending that diabetes care providers, like DEs, promote oral care among clients with diabetes, the evidence has identified that many have limited oral health knowledge, confidence, or health promotion resources to advocate for better oral health. As a result of these barriers, it appears oral health promotion is not integrated into the practice of many DEs. While oral health promotion intervention shave been developed for non-dental professionals in other countries (13), there is currently no comprehensive oral health training programs for DEs in Australia. Therefore, this study aimed to develop and evaluate a diabetes oral health training program to assess its potential impact in improving oral health knowledge and confidence of diabetes educators (DEs), and the acceptability, feasibility, and sustainability of integrating the training into practice. The findings from this preliminary evaluation will help to inform a future multi-centre randomised trial to examine the effectiveness of the intervention.

## 2 Methods

### 2.1 Design

Consistent with existing literature (14) a sequential mixed-methods, quasi-experimental one-group pretest-posttest design (15) was used to evaluate the DIOH training program. Evaluation of the program involved quantitative pre-post intervention questionnaires followed by qualitative interviews. The questionnaires explored changes in the oral health knowledge of DEs and their confidence in promoting oral health among clients. Through semi-structured interviews, the qualitative component investigated the perceptions of DEs on the acceptability, feasibility, and sustainability of integrating the intervention into clinical practice.

### 2.2 Framework

The Medical Research Council (MRC) framework for complex interventions underpins the development, evaluation and implementation of this overall program of study (16). The current study reports on the first and second steps of the MRC framework, *Developing Complex Interventions* and *Feasibility and Pilot*, respectively.

### 2.3 Setting and recruitment

The training program was evaluated among Australian DEs from various public health services in NSW and Victoria in Australia. DEs were recruited through a champion at the respective sites. The public health services were purposively selected to ensure a range of hospitals were represented. A total of four diabetes clinics across NSW (*n* = 3) and Victoria (*n* = 1) were included. The target sample size was selected based on a review of pilot and feasibility studies which identified that 30 participants (IQR: 20–45; range: 8–114) was the median sample size used in pilot studies (17).

Flyers and information sheets advertising the study were distributed via email to eligible staff. All interested DEs shared their e-mail address with the investigators to receive further information and the link to participate. All participants provided written consent. The champions supported initial recruitment and assisted with reminding DEs to complete the subsequent phases. There were no set exclusion criteria.

### 2.4 Developing the DIOH training program

A literature review was conducted to identify current gaps in this area and whether other oral health training programs had been developed. A multi-disciplinary working group was established to develop and evaluate the program. The planning process involved establishing research collaborations and partnerships with clinicians, academics, and policy makers from various disciplines (general practice, nursing and dentistry) and organisations (e.g., health services, universities and professional organisations) and consumers.

Feedback from diabetes care providers (DEs and GPs) identified the need for developing oral health promotion resources and an online e-learning workbook with a simple screening tool and referral pathways to oral health services (9, 10). Further, a survey among people with diabetes (18) showed the majority (84.0–89.0%) would participate in an oral health program delivered by diabetes care providers.

#### 2.4.1 Oral health promotion resources

After extensive consultation with the working group and consumers across NSW, an oral health promotion video and brochure were developed for people with diabetes. These resources were endorsed by the state government (NSW Health) and other key stakeholder including Diabetes Australia and Australian Dental Association and consumer groups. The video was uploaded to YouTube for easy accessibility for diabetes care providers and dental practitioners to promote oral health among people with diabetes (19) while the brochure was made available on the government website (12).

#### 2.4.2 E-learning workbook

The e-learning workbook was developed from an earlier version supported by the Australian Diabetes Educators Association (ADEA) (20). The workbook contained three sequential self-paced modules of approximately three-hour duration. Module 1 provided information about oral health and its relationship with diabetes and introduced how Diabetes Educators could play a role in oral health care. Module 2 provided a brief overview of the anatomy and physiology of the oral cavity, and potential changes that may occur in people with diabetes. Module 3 provided specific information to assist Diabetes Educators to promote oral health care in their practice including the oral health screening and referral process as well as a theoretical and practical skill assessment. The focus of the workbook was to build DEs' competence in providing oral health education, screening, and referral to people with diabetes.

**Figure 1:**
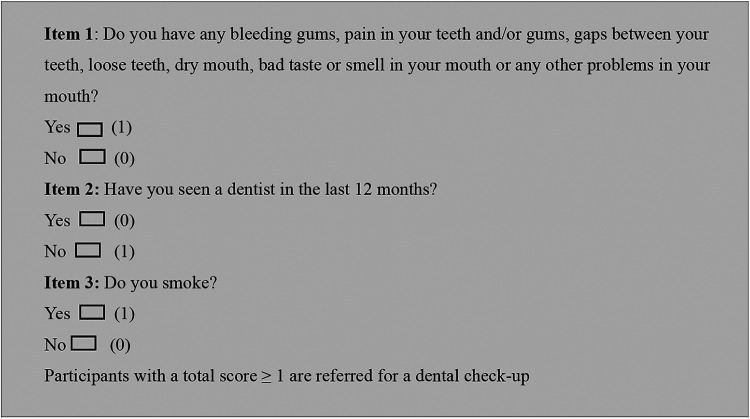
Oral health screening tool. Reproduced from “Oral health screening tool” by Ajesh George, Prakash Poudel, Ariana Kong, Amy Villarosa, Hanny Calache, Amit Arora, Rhonda Griffiths, Vincent W. Wong, Mark Gussy, Rachel E. Martin and Phyllis Lau, licensed under CC BY 4.0.

The workbook structure was modelled after a previous oral health training program for midwives, which was extensively evaluated (18, 21), endorsed nationally as a continuing professional development (CPD) activity (by the Australian College of Midwives) and recognised by the World Health Organisation (WHO) as an exemplary program (22). The information in the DIOH workbook was structured and written by clinicians, academics, and policymakers in the areas of oral health, diabetes, and translational research. Consumers who were DEs in the working group also reviewed the intervention. Various resources were included in the workbook including a previously validated, simple three-item oral health screening tool (high sensitivity, 93%) to identify people with diabetes at risk of poor oral health (Figure 1), state specific guidance on referrals to oral health services (15, 23) and oral health promotional material. A video showing how DEs could use the screening tool and other resources to integrate oral health into their practice was also included (24). The training was also made available as a printed workbook for the preliminary evaluation.

### 2.5 Data collection

Participating DEs were provided with a link or hard copy questionnaire, based on their preference. Participants were asked to complete a pre-intervention questionnaire comprising diabetes and oral health knowledge and confidence items. Recruitment commenced in August 2022, and the follow up was finalised in August 2023 when the study had recruited 30 participants.

The pre and post-intervention questionnaires were developed through a rigorous process involving a literature review to identify the latest evidence, a review by experts in the areas of oral health and diabetes, and pilot testing for face validity by experts and consumers (Supplementary File S1). Upon completing the pre-intervention questionnaire, DEs were provided login details to access the online DIOH education program. After completing the training, they completed the post-intervention questionnaire that included additional items exploring DEs' perceptions of the DIOH program. As the training was endorsed by the ADEA, DEs who completed the post-intervention questionnaire received a certificate to recognise the activity for three CPD hours.

DEs who completed the post-intervention questionnaire were also invited to participate in a semi-structured interview to discuss their feedback on the training around acceptability, feasibility, and sustainability (Supplementary File S2). The interviews were conducted through an online platform (e.g., Skype, Zoom) or over the phone and recorded (approximately 30–60 min). Participation in the follow-up interview was optional.

### 2.6 Data analysis

A sequential mixed-methods approach was utilised for data analysis, enabling researchers linking two datasets through a meaningful and sequential analysis (15). Quantitative data from the pre-post intervention questionnaires were analysed using the Statistical Package for Social Sciences (SPSS) software (Version 29.0). The total number of correct answers on the knowledge scale was calculated twice, before and after the completion of the intervention program. The oral health confidence was calculated based on the mean scores of the participants for each question in both the baseline and post-training questionnaires. The difference in the means of pre-test and post-test scores of knowledges and confidence was analysed for statistical significance using paired sample t-tests after checking for normality. The significance level for all analyses was set at *p* < 0.05. Additional exploratory subgroup analyses were conducted to explore baseline characteristics and its potential influence on the study outcomes.

The qualitative data from the audio recordings of the interviews were transcribed verbatim. All transcripts were then imported into the computer software NVivo to assist in coding and sorting segments of text. A directed content analysis was undertaken to analyse the qualitative questions (25). The analysis was based on prior research on oral health promotion pilot studies exploring the acceptability, feasibility and sustainability of the program (21). The codes were initially developed based on the framework, and the textual data was categorised based on the developed codes. Data that was not congruent to the existing categories were placed in newly generated categories. The data was then examined to determine whether codes needed to be sorted into categories or subcategories and compared the data to previous research in oral health promotion programs. The quantitative and qualitative data were integrated to address the study objectives where relevant.

## 3 Results

### 3.1 Demographics

A total of 30 DEs were recruited for the study but only 21 (16 from NSW and 5 from VIC) completed the post questionnaire and were included in the analysis. There were 3 DEs from VIC and 6 from NSW that were lost to follow up. Baseline demographic characteristics as well as knowledge and confidence scores were compared between participants who completed the study and those lost to follow up with no significant differences, although this should be interpreted with caution due to the small number of participants loss to follow up.

The mean age of the participants were 44.98 (±15.01) years, and the majority were females (85.7%). Of the total, 45.6% had a graduate certification or a diploma and around 23% had a master's degree. However, only 19% reported having received any education or training regarding oral health care for people with diabetes. Five participants from both NSW (*n* = 4) and Victoria (*n* = 1) participated in the interviews. Demographics of the participants are shown in Table 1.

**Table 1:** Demographics of the participants.

Characteristic	*N* = 21	(%)
Age in years; Mean (±SD)	44.98	±15.01
Gender		
Male	3	(14.3)
Female	18	(85.7)
Highest level of Education		
Bachelors	6	(28.6)
Graduate certificate/diploma	10	(47.6)
Masters	5	(23.8)
Years working as a Diabetes educator; mean(±SD)	9.51	±8.9
Received any education or training regarding oral health care for people with diabetes		
Yes	4	(19)
No	17	(81)
How often oral health is currently discussed with people with diabetes		
Frequently	3	(14.3)
Occasionally	10	(47.6)
Rarely	7	(33.3)
Never	1	(4.8)
Location of workplace		
Metropolitan and regional NSW	16	(76.2)
Metropolitan VIC	5	(23.8)

SD, standard deviation; NSW, New South Wales; VIC, Victoria.

### 3.2 Knowledge

Overall, there was a statistically significant increase in the total oral health knowledge scores after the DIOH training (Table 2). The participants had an increase in oral health knowledge, which was, on average, 6.43 points higher than at pre-training. The 95% CI around the difference is 4.23 and 8.62 points. The t-value of 6.11 with *p* < 0.001 confirms that the intervention significantly increased the knowledge scores. An additional subgroup analysis revealed that Diabetes Educators who had prior oral health training had a relatively higher baseline knowledge score compared to those who did not report prior training, although the post-test outcomes suggested that they were comparable following the training.

**Table 2:** Total number of knowledge questions completed correctly by participants.

Variable	Pre-test	Post-test	t (sig)
	Mean (SD) %	Mean (SD) %	
Knowledge score (score out of 35)	23.24 (4.40) 66.4%	29.67 (2.01) 84.8%	−6.11 (<0.001)
Received prior training (*n* = 4)	27.00 (2.94) 77.1%	29.25 (1.89) 83.6%	−2.64 (0.078)
No prior training (*n* = 17)	22.35 (4.27) 63.9%	29.76 (2.08) 85.0%	−6.35 (<0.001)

#### 3.2.1 Individual questions

A marked change (ranging from 4.0–52.0%) was observed in the knowledge scores post-intervention (Supplementary File S3). Topics where a measured improvement was observed included around the importance of encouraging clients to brush all surfaces of their teeth and tongue with soft brush and changing the toothbrush every six months, risk behaviours for periodontal disease (*Medication usage) a*nd awareness regarding current guidelines on the role of diabetes care providers (*Diabetes care providers need to identify oral health problems among clients*) (43.0–52.0% change). There was also knowledge improvement on the role of DEs in undertaking oral health screening and referrals (ranging from 10 to 33.0% change).

These findings were reiterated in the feedback from participants. Most (86.0%) agreed that the DIOH training had increased their awareness of the impact of oral health on diabetes management and that they understood the bidirectional link between oral health and diabetes (Supplementary File S4).

### 3.3 Confidence

After completing the education program, 83.0% of DEs felt that they were confident in discussing the topic of oral health with their clients. This was a significant increase in their confidence compared to before (52.0%) the training (t = −5.78, *p* < 0.001) (see Table 3). Furthermore, it was noted that the training resulted in a statistically significant increase in the confidence of DEs to conduct oral health screening (t = −5.59, *p* < 0.001) and refer clients to oral health services (t = −6.48, *p* < 0.001). An additional exploratory subgroup analysis suggested the small group of Diabetes Educators who received prior training reported higher pre-test confidence scores in discussing oral health and referring clients to dental services, although the scores were comparable to the group who did not receive prior training following the training program.

**Table 3:** Reported confidence level of participants.

Variable	Pre-test	Post-test	t-test (*p* value)
	Mean (SD) %	Mean (SD) %	
Confidence: DiscussingOH with clients	2.62 (0.973) 52.4%	4.14 (0.910) 82.8%	−5.78 (<0.001)
Received prior training (*n* = 4)	3.25 (0.96) 65.0%	4.00 (0.82) 80.0%	−3.00 (0.058)
No prior training (*n* = 17)	2.47 (0.94) 49.4%	4.18 (0.95) 83.6%	−5.57 (<0.001)
Confidence: Screening for OH problems	2.67 (1.15) 53%	4.19 (0.928) 84%	−5.59 (<0.001)
Received prior training	2.50 (0.57) 50.0%	4.25 (0.96) 85.0%	−1.57 (0.215)
No prior training	2.47 (1.18) 49.4%	4.18 (0.95) 83.6%	−5.57 (<0.001)
Confidence: Referring clients to oral services	2.52 (1.12) 50%	4.05 (0.865) 81%	−6.48 (<0.001)
Received prior training	3.00 (1.15) 60.0%	4.25 (0.96) 85.0%	−1.99 (0.141)
No prior training	2.41 (1.12) 48.2%	4.00 (0.87) 80.0%	−6.15 (<0.001)

(Response scale of 1–5: where 1 = “not confident at all” and 5 = “completely confident”).

Within the qualitative interviews one participant mentioned noticing a change in their confidence after training. This participant recalled, “*I feel more confident..”* (DE_4) and “(it) *helped me to..* *I suppose, (be) adept at those more technical aspects.*” (DE_4, 4 yrs experience).

### 3.4 Acceptability of undertaking the training

Regarding the training content, 91% of the participants agreed that the content was easy to understand and relevant to their work. About 95% felt the screening tool was easy to use, and 90% would recommend the training to other colleagues (Supplementary File S4).

During the qualitative interviews, participants provided feedback about the e-learning workbook, supporting resources (screening tool and oral health promotional material), and access to the training.

### 3.5 E-learning workbook & supporting resources

All participants provided positive feedback on the content and delivery of information within the workbook. Participants commented that “*I think it's a pretty comprehensive booklet..* *it didn't take me that long to do” (DE_1, 9 yrs experience)* and, felt that it provided a quick overview of the topic: “*refreshing our knowledge about our resources, especially in line with our profession* “(DE_2, 3 yrs experience). Some participants appreciated the use of images throughout the workbook. These participants reported that the images enhanced their knowledge and awareness by contrasting and comparing oral health vs. oral disease.

“…its well presented because there is a picture as well… When we talking about a healthy gum, What is the healthy gum? So with the pictures that we can see better.. It's a lot of picture color, less word, more picture..will attract more with the picture rather than very lengthy words, so I think it's very well presented here.” (DE_3, 3 yrs experience)

Overall, the sentiment was that the training was not burdensome to complete, with one participant reporting on their experience of participating in the training session, requiring a couple of hours of commitment:

“Answering all the activities, I suppose if you just read it, yeah, it would be a little bit of time, but I would say a couple of hours at least. Couple of hours.” (DE_5, 3 yrs experience)

Some participants acknowledged that the information within the booklet was presented in a practical format and “*delivered in a method that everybody can understand*” (DE_3, 3 yrs experience). A few participants also commented on the relevance of the information provided to their clinical profession.

The screening tool was another aspect of training that was well received by all participants and something that they considered easy to use. Comments from some participants regarding the ease of use and acceptability of the screening tool included: “*I was pleasantly surprised..*” (DE_4, 4 yrs experience), “*Questions were easy..*” (DE_5, 3 yrs experience), and “*I think it's a good tool.*” (DE_1, 9 yrs experience).

Several participants highlighted that the screening tool was “*nice and succinct and straightforward*” (DE_4, 4 yrs experience) and it could be delivered and used as a guide during consultation with the patient:

“Sometimes you probably want (to) do it in the exact order because a lot of these would cut through the conversation. But yeah, I think that its pretty good and it has some guidance as well of what to do, where the patient to go” (DE_5, 3 yrs experience)

The same participant also commented on the neutrality of the screening questions within the screening tool and how this enabled the health provider to not come across as judgmental or biased.

“I think good questions and.. then not, how can I put it, aggressive questions or so personal questions. They're quite normal questions.” (DE_5)

### 3.6 Accessing the training

Few participants commented on the accessibility of the training. Although the layout and inclusion of resources were seen as valuable, the placement of resources at the back of the e-workbook led to forgetfulness about their existence, often asking: *Is that in this booklet?* (DE_1, 9 yrs experience).

Regarding the workbook, some participants appreciated that they could complete the workbook both online, and via hard copy due to the limited availability of computers.

“I think that the good thing was you could do it on the book or online because a lot of times we don't have a computer in front of us. That was pretty handy.” (DE_5, 3 yrs experience)

Other participants also highlighted that the content was delivered in a method that was “*quite easy to follow*.” (DE_5, 3 yrs experience) and was not considered time consuming: “ *it didn't take me that long to do it, which was good*.” (DE_1, 9 yrs experience)

### 3.7 Feasibility of integration training into practice

Of the total, 91% of participants agreed that their knowledge of oral health had improved due to the training and that they would discuss oral health with their clients (Table 4).

**Table 4:** Participants feedback on the DIOH training content .

Feedback on results of this training	Range	Mean (SD)	% of those who agreed to the statements
My knowledge of oral health has improved.	3.00–5.00	4.57 (.68)	91
I will discuss oral health with clients.	3.00–5.00	4.57 (.68)	91
I will refer clients for regular, oral health check-ups.	3.00–5.00	4.43 (.75)	86

(Response scale of 1–5, where 1 = strongly disagree, 5 = strongly agree).

Participants reported that integrating the screening tool into their usual practice would be straightforward as “*they were simple questions and questions that we always ask “*(DE_5, 3 yrs experience). Further, it was also thought that it had the potential for future implementation in their practice: “*certainly something that we will look at implementing*” (DE_4, 4 yrs experience)

Another participant commented on a change in their perceptions toward oral health and how they prioritised it now in their practice. Following training, this participant noticed that the topic of oral health was now something they included in all their conversations with clients:

“… when I'm talking to patients that umm, that is [not] one of the top lists that in my head that I should always discuss it. But now that after I've done the training, I have always talk about, you know, brushing your teeth twice a day, …the routine that you should do as looking after yourself.” (DE_3, 3 yrs experience)

### 3.8 Sustainability of program

A few participants explored the sustainability of integrating the oral health training into their practice, specifically, the logistics of including the screening tool. Although the screening tool was well received, a third of participants identified a few challenges to using the paper-based form and discussed on the potential to digitise the tool:

“it's just, again, another piece of paper that I don't automatically have with you…” (DE_1, 9 yrs experience)

“Our system [electronic medical record system] probably wouldn't support something like that being included in it. So it would have to be at this point. Something an addition, you know..” (DE_4, 4 yrs experience)

Some participants suggested that this could be solved by “print[ing] off a heap of the oral health screening tools and have them sitting in the room, and then that will prompt people to use them.” (DE_1, 9 yrs experience)

Two participants identified some minor concerns when integrating the training program into clinical care; specifically, the time constraints to including oral health. Two participants discussed the challenge of managing other priorities in practice:

“not very, very often [talking about oral health in practice]…due to time constraints, we have other priorities.” (DE_2, 3 yrs experience)

“It's probably not the priority when people come to see, they come see for lots of other things..” (DE_1, 9 yrs experience)

Many participants provided suggestions for how the training program and associated resources (e.g., tool), could be broadened to ensure that material was accessible, easily integrated, and met the needs of their clients. Some participants discussed and made the suggestions regarding making resources available on public platforms such as *Go Share* (content distribution platform) and key diabetes bodies such as *Diabetes Australia:*

“Go Share, is a platform…basically, they try to locate all the relevant resources that I might be around type 1, type 2 diabetes… there's lots of little videos and stuff that have been developed specifically for. But I also go to Diabetes Australia, NDIS [National Disability Insurance Scheme], International Diabetes Institute in Victoria…put those resources that they have up [on Go Share].. So the idea, of course, is that I don't have to go to NDIS (India) or Australia – I can just go to Go share.” (DE_4, 4 yrs experience)

Another participant also suggested distributing the resources (screening tool and oral health promotional resources) to ensure that other staff, such as nurse practitioners, who may be working with clients with diabetes, and have access to these oral health resources:

“I think it probably needs to be accessible to practice nurses and most practice nurses aren't credentialed DEs, so they won't have access to it. So probably some, some, some way of accessing information through Diabetes New South Wales, like those factsheets, would be helpful..” (DE_1, 9 yrs experience)

Some participants also discussed including more information on the impact of nutrition on oral health and reproducing resources in different languages for individuals from culturally and linguistically diverse backgrounds:

“Particularly around nutrition and healthy eating.. we are focused very much on the nutritional aspects of this.” (DE_4, 4 yrs experience)

“..I'm hoping also a lot of those going to a different language as well for people that is non English-speaking background.” (DE_3, 3 yrs experience)

It was also discussed that clients may benefit from having the screening tool incorporated into the routine annual diabetes health check list with their general practitioner as a prompt for encouraging preventative oral health care:

“..the annual cycle of care is stuff that we go through with patients..they do it with their GP to check that they're doing everything to sort of manage their diabetes,.. So, I think if dentistry was on there, it would prompt people to go, Oh, you haven't made it into it in 12 months.” (DE_1, 9 yrs experience)

## 4 Discussion

An evidenced-based strategy to incorporate oral health into primary care includes the integration of oral health into the services of primary care providers through education and training, as well as policy changes (26). Among diabetes care providers, the need for oral health training is important particularly due to the documented knowledge gaps regarding the bidirectional link between diabetes and periodontal disease (9, 11, 27) and the improved glycaemic control following periodontal treatment (6).

The findings from this preliminary evaluation of the DIOH training program demonstrated a statistically significant increase in oral health knowledge and confidence among DEs. There were measured improvements in participants' knowledge of the risk factors that could exacerbate periodontal disease risk. Recognising other risk factors, such as cardiovascular disease which can also independently increase the risk of periodontal disease (28), is important because of the prevalence of comorbidities among people with diabetes. Since many people with diabetes take medication (29), it is notable that all participants understood the risk between medications and xerostomia. Additionally, they recognised the significance of encouraging clients to engage in regular oral hygiene practices. Sub-optimal oral hygiene has been associated with a two-to-fivefold increase in periodontitis (30). While most of the studies included in this meta-analysis (30) were of lower quality, it nevertheless demonstrates that diabetes care providers could promote oral health by reminding clients to engage in regular oral hygiene practices. In our findings, there was a statistically significant increase seen across DEs' confidence in discussing, screening and referring clients to oral health services, which was also reflected in the qualitative feedback.

Translating knowledge into clinical practice requires an examination of various barriers, as well as the acceptability, feasibility and sustainability of implementing the intervention into a service (31). The DEs found the training program, including the screening tool, to be both acceptable and feasible as it was notably clear, short, and simple. The DEs' confidence and acceptability in using the screening tool is important because of its usefulness in triggering referrals to the oral health services for follow-up, which is a critical component in improving oral health integration (9, 26). It should be noted that referral pathways to dental services differ depending on the area, and part of this program included awareness of the requirement for individual adaptation of referral pathways depending upon the clients' individual risk level, their location, and their eligibility for public dental services.

To our knowledge, our study is the first to demonstrate a statistically significant increase in oral health knowledge and confidence among diabetes care providers in screening clients using online, interactive education modules while also being certified to credit continuing professional development points from the national DE association. Past studies on training diabetes care providers in oral health have used various modalities to deliver training, including live or video lectures in the Pacific Islands (32), pre-recorded modules delivered via a computer-based format (32), or in-person workshops and clinical simulations in the United States (33, 34). In the study by Chen, Buenconsejo-Lum (32), the participants appreciated the computer-based format, but only the groups who watched the live or video broadcast of the lecture had a significant increase in oral health knowledge. The other study by Dounis, Ditmyer (33) did not examine knowledge relating to diabetes and oral health, nor their confidence in screening and referrals. Another American study by Kordsmeier, Ty Williams (34) evaluated a nurse practitioner-led oral health program to increase the oral health knowledge of medical assistants through in-person training. The authors also adapted a five-item oral health screening tool from the National Health and Nutrition Examination Survey (35). While the training increased oral health knowledge, and the screening tool was found to be largely acceptable, participants commented that some dental patients were unsure how to complete the screening questions (34).

Mitigating barriers that limit the support for integrating oral health into primary care is vital for the long-term sustainability of this model. Such barriers from the DIOH training program include time constraints, limited efficiency with a paper-based screening tool. Time constraints leading to low prioritisation to discuss oral health is a well-documented barrier to integration (36), and is likely why participants highlighted the need for oral health promotion to be part of the role of the entire diabetes clinical team. Another major barrier is that a significant proportion people with diabetes never receive diabetes education or see a diabetes nurse educator (35). Thus, future research and programs should adapt and disseminate training to the broader clinical team, including dietitians, general practitioners, and endocrinologists. While the screening tool was convenient, seamless and efficient integration of the tool as well as automated oral health referral letters for clients would require a modification in the electronic medical record system. This strategy has been previously proposed in an earlier study (34).

New South Wales has limited specific public oral health referral pathways for people with diabetes (37). In Victoria, medical reasons such as diabetes are not considered a reason for “priority care” in public dental services, hence people with diabetes, who may be eligible for public dental care and unable to afford care in a private dental service, may find themselves waiting for up to 3 or more years on a public dental waiting list before receiving dental care. The gap in public oral health policy may be because more conclusive, substantiated evidence stating that periodontal treatment can improve HbA1c by a clinically significant amount has only been published recently (6). Simpson, Clarkson (6) advocated that future research should focus on integrating oral health care into diabetes management by coordinating dental and non-dental care providers.

Changes in public preventative oral health policy, especially periodontal advice and care for people with diabetes, should be influenced by the evidence that shows a reduction in the expenditure of managing diabetes-related complications. A few studies in Europe and the United States reported that providing periodontal treatment to individuals with diabetes reduced the cost of diabetes-related health care. Smits, Listl (38) calculated that in the Netherlands, the overall reduction in expenditure for diabetes-related healthcare costs per patient per quarter of the year was €12. These reductions in cost were seen particularly among individuals receiving insulin therapy. In the United States, Thakkar-Samtani, Heaton (39) found that among people with diabetes who received periodontal treatment and had public or private health insurance, there was a 14% and 12% reduction in overall healthcare costs, respectively, compared to people who did not receive periodontal treatment. Comparatively, a German study found that periodontal treatment provided a small but statistically insignificant reduction in healthcare costs among newly diagnosed people with diabetes (40). One reason for these significant cost savings in healthcare may be due to the associated risk between periodontal disease and other diabetes-related micro- and macrovascular complications (41). In Australia, an economic evaluation would need to be conducted to determine the cost-effectiveness of introducing periodontal therapy among people with diabetes within public oral health services and subsequently shape public oral health policy.

Although this study included DEs working from several selected clinics in New South Wales and Victoria, the sample size was small especially for the follow up interviews, which limits data validity. This loss to follow up is reflected in an Australian study which found that engagement of research activities among DEs is low due to a lack of time and resources (1). Nevertheless, a previous review on pilot and feasibility studies identified that 20–50 participants was in the interquartile range of pilot and feasibility studies (17). Further, as this study was a preliminary evaluation, it only measured short-term outcomes and would require further research to explore sustained clinical practice adaptation of DEs, as well as the impact of implementing the screening and referral process onto the electronic medical record system. This quasi-experimental study did not control for several participant-level factors, including years of professional experience, prior exposure to interdisciplinary care, baseline attitudes towards oral health and personal motivation, which may have influenced study outcomes. Prior oral health training among a subset of participants may have acted as a confounding factor, influencing both baseline knowledge and responsiveness to the intervention. This may have contributed to variability in outcomes and should be considered when interpreting the findings. Future studies with larger samples should account for such factors through stratified or adjusted analyses. In addition, the study design may have been susceptible to testing effects, social desirability bias and other external influences. Sample attrition may have influenced the study findings, as participants who completed the intervention may have been more motivated or engaged than those who withdrew. This could have resulted in an overestimation of the observed improvements, limiting the generalisability of the results. Future studies should employ more rigorous study designs with larger sample sizes control for potential confounders such as prior oral health training. A larger sample size would also help to address challenges with recruitment and loss to follow up.

## 5 Conclusions

This study has demonstrated a preliminary impact of an online training program with tailored resources in improving the oral health knowledge and confidence of DEs. The integration of the training into DEs' clinical practice was demonstrated as acceptable and feasible; however, a more rigorous design such as a randomised controlled trial is needed to establish causal inference. For long-term sustainability, future research would need to focus on the long-term effectiveness of the training and administering the training to other diabetes care providers while maintaining the same incentive for professional development. We recommend additional evaluation of the program which will eventually inform a dissemination strategy with stakeholders across Australia to promote this CPD program among DEs, which would include distributing oral health promotion resources from the program with all stakeholders involved in the care of people with diabetes. The findings from this research has provided a valuable platform to build on and eventually inform national public health policy on holistic preventive oral health care for individuals with diabetes, leveraging on national efforts to maximise the use of patients' single digital health records.

Public health policy changes should also investigate integrating the screening tool and referral processes into the electronic medical record system. The economic value of giving periodontal therapy to individuals with diabetes could be considered as a priority area for policy and research in Australia, with increasing evidence internationally of the impact of periodontal care on reducing the overall cost of diabetes-related health care. Further testing of DIOH training program and assessing its effectiveness in improving oral health outcomes and glycaemic control in Australia would greatly support future policy change in this area.

## Data Availability

The raw data supporting the conclusions of this article will be made available by the authors, without undue reservation.
