# Supplementary figures and images for: Development and preliminary evaluation of an oral health training program for diabetes educators: a quasi-experimental study
Source: Front Oral Health. 2026 May 21;7:1819829. doi: 10.3389/froh.2026.1819829 (PMC13233408; doi:10.3389/froh.2026.1819829)

# Supplementary File 1: Pre and Post Questionnaires


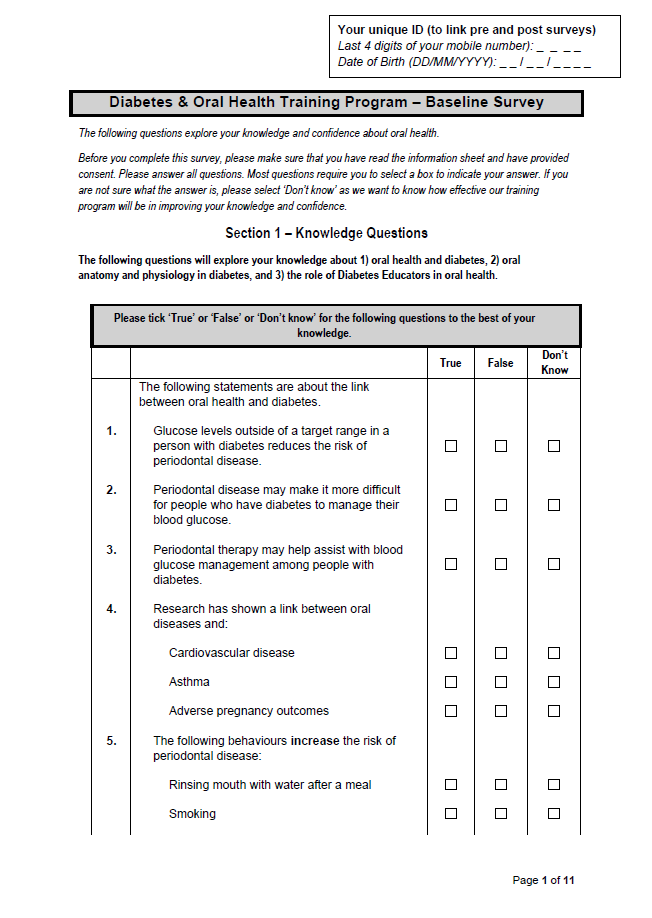


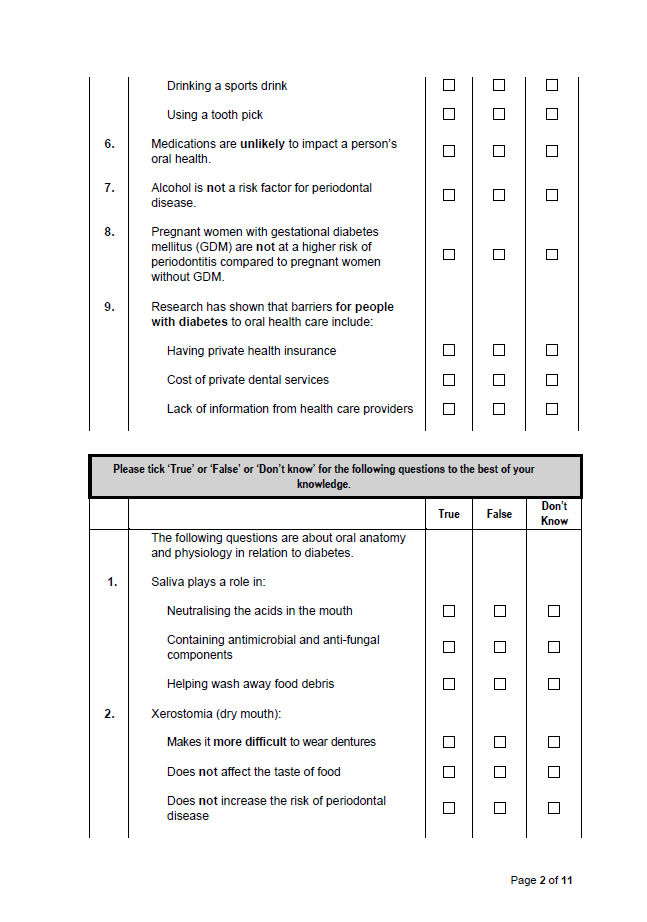

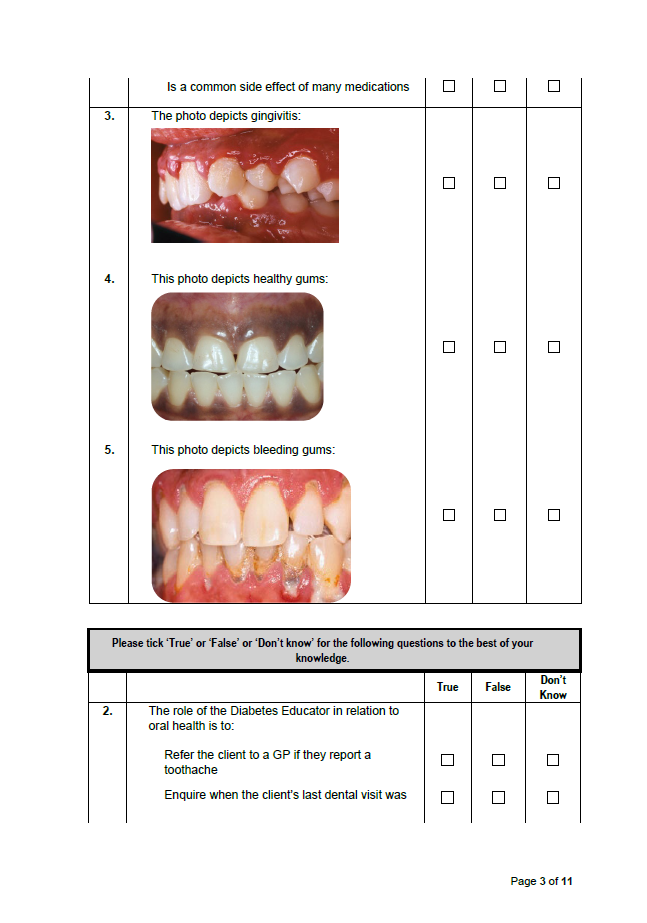

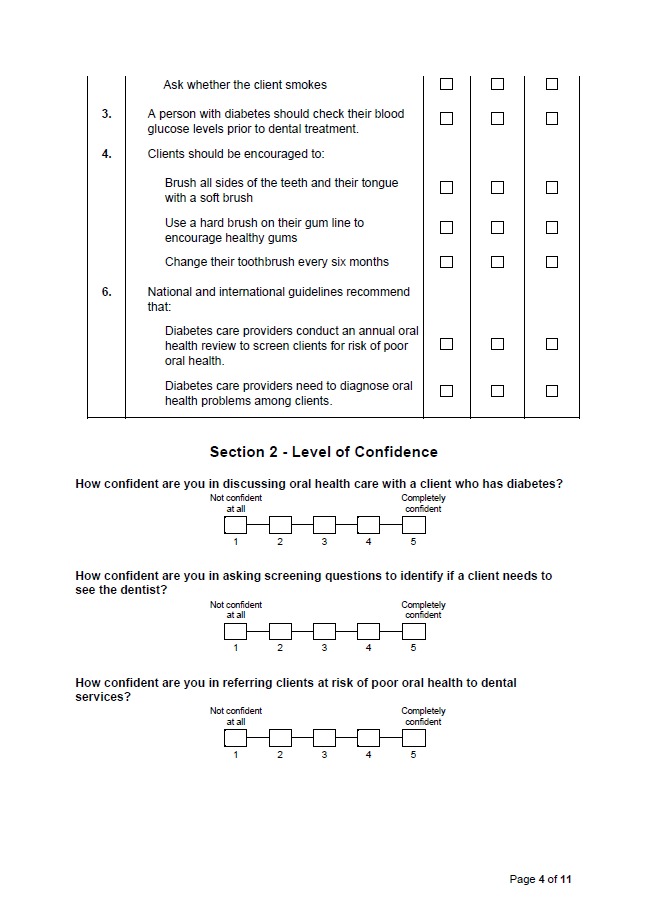

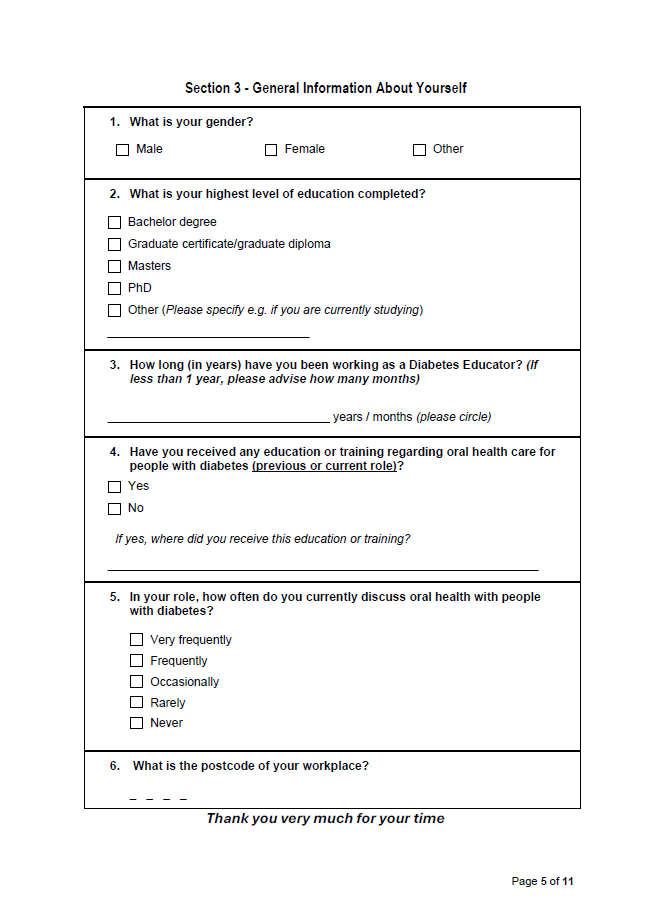

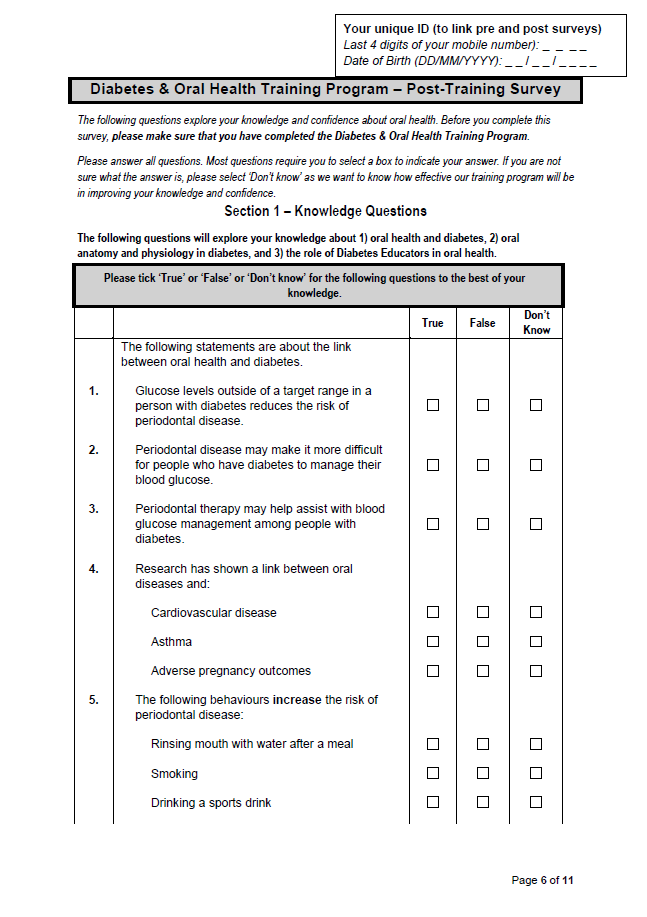

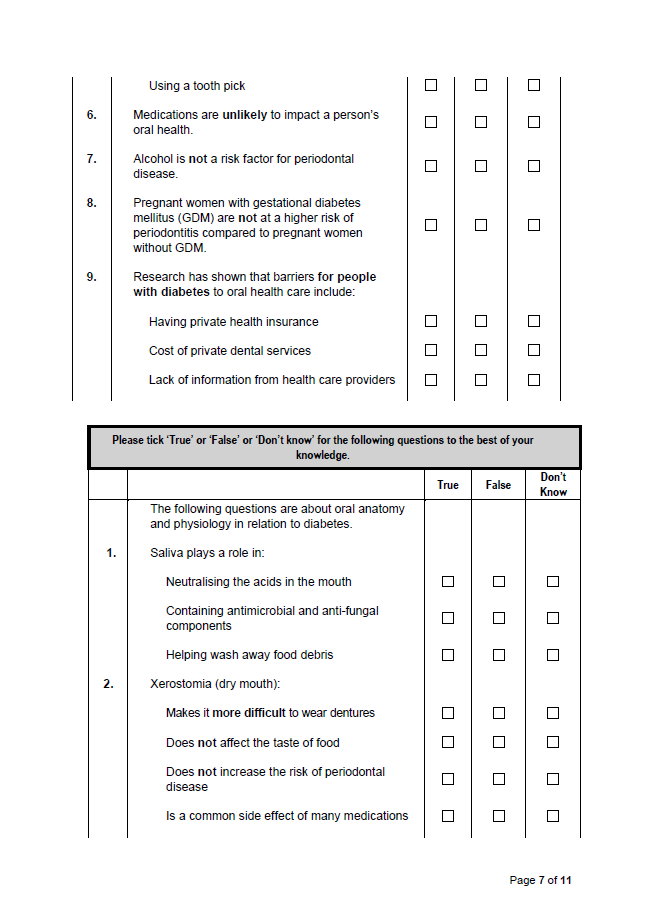

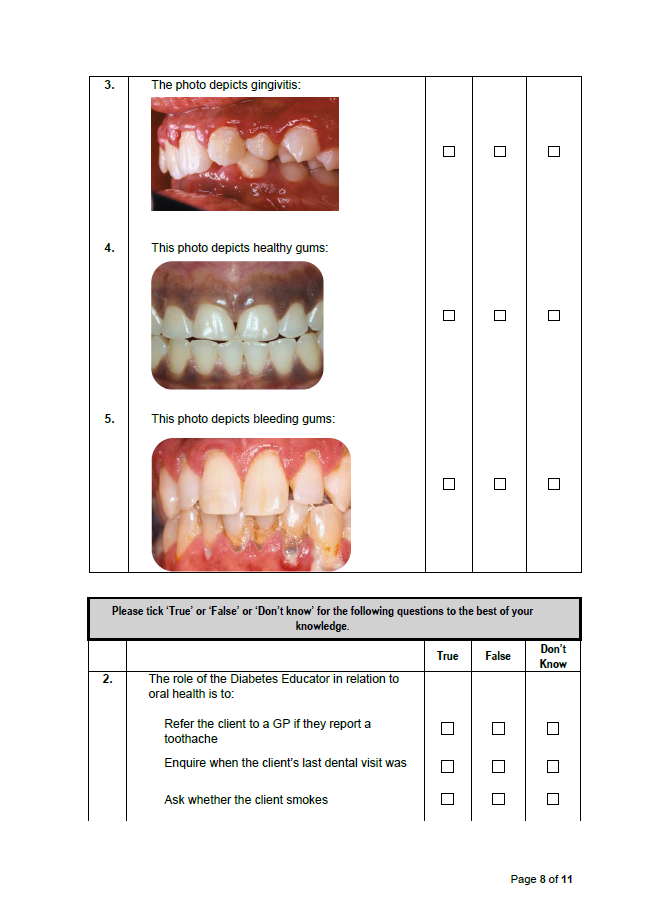

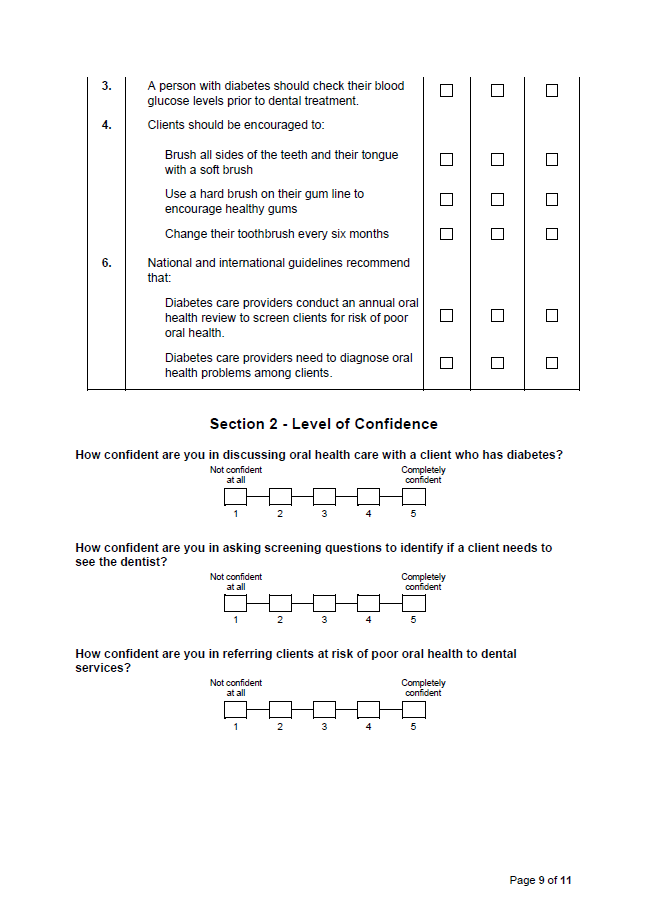

Supplement: Supplementary file 1 [file Supplementaryfile1.docx]
