# Supplementary material for: Development and preliminary evaluation of an oral health training program for diabetes educators: a quasi-experimental study
Source: Front Oral Health. 2026 May 21;7:1819829. doi: 10.3389/froh.2026.1819829 (PMC13233408; doi:10.3389/froh.2026.1819829)
Supplement: Supplementary file 2 [file Supplementaryfile2.docx]

# Supplementary File 2: Interview topic guide

Topic Area 1: Acceptability of the training program

- Perceived role of Diabetes Educators in oral health
- Content & pitch of the training (appropriate, useful, relevant)
- Appropriateness of the screening tool

Topic Area 2: Feasibility of integrating the training into practice

- Self-perceived change in oral health knowledge and confidence
- How would they integrate oral health into practice?
  - [If difficult]: what additional supports are needed?
- Enablers and barriers towards implementation (e.g. time constraints)

Topic Area 3: Sustainability of a model of care

- Dental referrals: ideally, what would this look like? (e.g. interaction with oral health professional)
- Ease of linking clients to dental services (public, private, Aboriginal community controlled health services)
- Any recommendations to ensure oral health can be sustainably implemented in long-term practice
