# Supplementary material for: Development and preliminary evaluation of an oral health training program for diabetes educators: a quasi-experimental study
Source: Front Oral Health. 2026 May 21;7:1819829. doi: 10.3389/froh.2026.1819829 (PMC13233408; doi:10.3389/froh.2026.1819829)
Supplement: Supplementary file 3 [file Supplementaryfile3.docx]

# Supplementary File 3: Correct responses given by participants to individual knowledge questions pre and post-DIOH training

| ***Questions*** | ***Pre N (%)*** | ***Post N (%)*** | ***% change*** |
| --- | --- | --- | --- |
| Glucose levels outside of a target range in a person with diabetes reduces the risk of periodontal disease. | 16 (76) | 21 (100) | +24 |
| Periodontal disease may make it more difficult for people who have diabetes to manage their blood glucose. | 21 (100) | 21 (100) | - |
| Periodontal therapy may help assist with blood glucose management among people with diabetes. | 18 (86) | 21 (100) | +14 |
| *Research has shown a link between oral diseases and:* |  |  |  |
| Cardiovascular disease | 16 (76) | 21 (100) | +24 |
| Asthma | 5 (24) | 8 (38) | +14 |
| Adverse pregnancy outcomes | 10 (48) | 18 (86) | +38 |
| *The following behaviours increase the risk of periodontal disease:* |  |  |  |
| Rinsing mouth with water after a meal | 17 (81) | 20 (95) | +14 |
| Smoking | 21 (100) | 21 (100) | - |
| Drinking a sports drink | 21 (100) | 21 (100) | - |
| Using a toothpick | 9 (43) | 9 (43) | - |
| Medications are unlikely to impact a person’s oral health. | 12 (57) | 21 (100) | +43 |
| Alcohol is not a risk factor for periodontal disease. | 14 (67) | 19 (90) | +23 |
| Pregnant women with gestational diabetes mellitus (GDM) are not at a higher risk of periodontitis compared to pregnant women without GDM. | 13 (62) | 21 (100) | +38 |
| *Research has shown that barriers for people with diabetes to oral health care include:* |  |  |  |
| Having private health insurance | 6 (29) | 7 (33) | +4 |
| Cost of private dental services | 19 (90) | 21 (100) | +9 |
| Lack of information from health care providers | 16 (76) | 21 (100) | +24 |
| *Saliva plays a role in:* |  |  |  |
| Neutralising the acids in the mouth | 18 (86) | 21 (100) | +14 |
| Containing antimicrobial and anti-fungal components | 16 (76) | 21 (!00) | +24 |
| Helping wash away food debris | 19 (90) | 21(100) | +10 |
| *Xerostomia (dry mouth):* |  |  |  |
| Makes it more difficult to wear dentures | 19 (90) | 21 (100) | +10 |
| Does not affect the taste of food | 17 (81) | 20 (95) | +14 |
| Does not increase the risk of periodontal disease | 17 (81) | 21 (100) | +19 |
| Is a common side effect of many medications | 19 (90) | 21 (100) | +10 |
| *The role of the Diabetes Educator in relation to oral health is to:* |  |  |  |
| Refer the client to a GP if they report a toothache | 5 (24) | 12 (57) | +33 |
| Enquire when the client’s last dental visit was | 20 (90) | 21 (100) | +10 |
| Ask whether the client smokes | 21 (100) | 21 (100) | - |
| A person with diabetes should check their blood glucose levels prior to dental treatment. | 18 (86) | 20 (95) | +9 |
| *Clients should be encouraged to:* |  |  |  |
| Brush all sides of the teeth and their tongue with a soft brush | 10 (48) | 21 (100) | +52 |
| Use a hard brush on their gum line to encourage healthy gums | 17 (81) | 19 (90) | +9 |
| Change their toothbrush every six months | 5 (24) | 12 (57) | +33 |
| *National and international guidelines recommend that:* |  |  |  |
| Diabetes care providers conduct an annual oral health review to screen clients for risk of poor oral health. | 15 (71) | 21 (100) | +29 |
| Diabetes care providers need to diagnose oral health problems among clients. | 5 (24) | 14 (67) | +43 |
