# Supplementary material for: Development and preliminary evaluation of an oral health training program for diabetes educators: a quasi-experimental study
Source: Front Oral Health. 2026 May 21;7:1819829. doi: 10.3389/froh.2026.1819829 (PMC13233408; doi:10.3389/froh.2026.1819829)
Supplement: Supplementary file 4 [file Supplementaryfile4.docx]

# Supplementary File 4a: Participants feedback on the DIOH learning objective

(Response scale of 1-5, where 1 = strongly disagree, 5 = strongly agree)

| **Learning Objective** | **Range** | **Mean (SD)** | **% of those who agreed to the statements** |
| --- | --- | --- | --- |
| I have an increased awareness of the impact of oral health on diabetes management. | 3.00 - 5.00 | 4.48 (.75) | 86 |
| I understand the bi-directional link between oral health and diabetes. | 3.00 - 5.00 | 4.48 (.75) | 86 |
| I recognise additional factors that predispose poor oral health and barriers to oral health care. | 3.00 - 5.00 | 4.38 (.80) | 81 |
| I can identify differences between a normal, healthy mouth and one that may be unhealthy. | 3.00 - 5.00 | 4.33 (.80) | 81 |

# Supplementary File 4b: Participants feedback on the DIOH training content

(Response scale of 1-5, where 1 = strongly disagree, 5 = strongly agree)

| *Training content:* | **Range** | **Mean (SD)** | **% of those who agreed to the statements** |
| --- | --- | --- | --- |
| The content was easy to understand. | 3.00 - 5.00 | 4.52 (.68) | 91 |
| The material was relevant to my work. | 3.00 - 5.00 | 4.57 (.68) | 91 |
| The screening tool is easy to use. | 3.00 - 5.00 | 4.52 (.60) | 95 |
| The referral pathways would be appropriate to use. | 3.00 - 5.00 | 4.38 (.67) | 91 |
| The length of the training was adequate. | 3.00 - 5.00 | 4.43 (.68) | 91 |
| I would recommend this training to a colleague. | 3.00 - 5.00 | 4.48 (.68) | 90 |
